# Foundational Aspects of Neurology Education Research: Philosophy of Science as the Starting Point of Neurology Education Research

**DOI:** 10.1212/NE9.0000000000200177

**Published:** 2024-12-30

**Authors:** Andres Fernandez, Lara Varpio

**Affiliations:** 1 Department of Neurology, Thomas Jefferson University, Philadelphia, PA;; 2 School of Health Professions Education (SHE), Maastricht University, the Netherlands;; 3 Perelman School of Medicine, University of Pennsylvania, Philadelphia; and; 4 The Children's Hospital of Philadelphia, PA.

## Abstract

This article provides a discussion on foundational aspects of neurology education based on concepts from the philosophy of science: ontology, epistemology, and paradigms. Together, these concepts are the starting point of the education research process. We provide context for the application of these concepts by outlining how they are cornerstones of high-quality neurology education research and by describing the underlying differences between education research and biomedical research. We illustrate these concepts through 2 case scenarios and offer practical considerations for planning neurology education research.

The creation of the new journal—*Neurology*® *Education*—has provided a venue to publish neurology education research. Still, neurology education research lags behind other specialties (e.g., for decades, the *Journal of General Internal Medicine* has had an entire section of the editorial board devoted to handling the many medical education–focused studies). To promote the role that education plays in the practice of neurology, to meaningfully advance the understanding of how to influence care through teaching and learning, and to create evidence-informed educational practices, we must take on the challenge of engaging in high-quality neurology education research.

The knowledge, skills, and attitudes (KSAs) that each neurologist wields are significantly shaped by the quality of the education they receive. By extension, then, the quality of the patient care that neurologists deliver in practice is substantially formed by their education experiences. To shore up the KSAs of these future practitioners, time and energy are dedicated to develop neuroscience courses, constructing neurology clerkship instruction for medical students and attending to the workplace-based learning of residents and fellows. Curricula are built to foster supportive learning environments and develop assessments to prepare learners for practice. Through this work, the course is set for the next generation of neurologists. Acknowledging the impact that neurology education can have in shaping future neurologists highlights the need to develop high-quality neurology education research. Exploring concepts such as *teaching approaches* and *learning needs* through neurology education research will serve as the foundation for developing higher quality education for the field with the goal to ultimately affect care and outcomes.

Participation in neurology education research should reach beyond basic descriptions of educational initiatives implemented at individual centers; instead, it should strive to produce high-quality research that joins and broadens a scholarly conversation in the education research literature. Advancing neurology education requires a conversation about how to do high-quality education research. Performing education research is a process with many steps; this article provides a discussion of the initial steps based on concepts from the philosophy of science: ontology, epistemology, and paradigms. Neurology education researchers should have familiarity with these concepts when starting an educational research project. Future articles in the Fundamentals of Neurology Education Research (FuNER) series will build on these concepts to outline their influence in other parts of the research process (including their influence in the methodology, methods, and research design).

To provide context for thinking about the ontology, epistemology, and paradigms underpinning neurology education research, we will discuss the importance of education research in neurology and how it fundamentally differs from the biomedical research models. We will use these terms to illustrate how different kinds of neurology education questions can be addressed from different paradigms (each with its own ontology and epistemology).

## Definition of Key Terms

- Ontology is defined as the nature (the reality) of the phenomenon to be studied (single generalizable truth to the phenomenon vs multiple truths where what is determined to be “real” is different between contexts).
- Epistemology is the kind of knowledge about that phenomenon that is developed through the research.
- Ontology and epistemology are constituent elements of a paradigm. A paradigm is “the set of common beliefs and agreements shared between scientists about how problems should be understood and addressed.”^1^ A paradigm “consists of the concepts, practices, and language that define a particular approach to science.”^2^ In other words, a paradigm is comprised of the foundational assumptions about how to conduct good research.

The Table provides a detailed description of the abovementioned key terms and their applications based on the case scenarios described further.

**Table:** Description and Application of Key Terms

Key term	Description/definition	Questions that underpin the key term	Application in the example scenarios
Paradigm	Specific set of foundational assumptions about the phenomena we are studying	What is the perception of the nature of reality and the nature of knowledge about the phenomenon being studied? (ontology and epistemology mentioned further can be referred as elements of the paradigm)	Scenario 1 (lumbar puncture competence): Post-positivist paradigm: • Ontology: there is an objective, tangible, and measurable reality that can be inferred by observation but understanding of the reality is incomplete or probabilistic • Epistemology: knowledge is progressive, and our understanding of the phenomena evolves with new research Scenario 2 (communicating a new neurologic diagnosis): Constructivism paradigm: • Ontology: multiple subjective realities exist that are socially and empirically based • Epistemology: knowledge is constructed; it is the product of interpretation and is context-specific
Ontology	Nature of reality (nature of the phenomena we are studying with our research)	Is there a single generalizable truth to the phenomenon we are studying, or are there multiple truths where what is determined to be “real” and true about the phenomenon is different between different contexts?	Scenario 1 (lumbar puncture competence): • There is a physical reality that we can objectively observe to construct cause-and-effect relationships • Lumbar puncture skills can be labeled, defined, measured, and assessed Scenario 2 (communicating a new neurologic diagnosis): • The reality of how best to communicate a new diagnosis is a social and experiential construction • There is no single best (or right) way to engage in this communication because what might be effective communication expectations for some will stand in direct contradiction of the expectations held by others
Epistemology	Nature of knowledge that can be developed through the research	What type of knowledge can we generate with our research study?	Scenario 1 (lumbar puncture competence): • We want to develop unbiased knowledge about the procedure and the skills • The knowledge should be generalizable so that the expected skills would be the same across different contexts Scenario 2 (communicating a new neurologic diagnosis): • We can develop knowledge about what it is like for a specific community or group to receive a new diagnosis. In so doing, we can identify principles and considerations that can make the communication of a new diagnosis “better” for *this* population. These principles and considerations might be transferable to other populations that are sufficiently similar in important kinds of ways • The research will generate rich, detailed insights about the experiences and perspectives of the specific patients in the study

The content for the Table is based on references 2, 7, and 8.

To bring these key terms together, consider a common educational situation: an attending physician is having a corridor conversation with a junior resident to educate this learner. *Ontology* refers to how we consider the nature of the conversation. Is there a “right” way to have this conversation (i.e., is there an ideal version of this conversation that should be sought)? Is there a single, real version of this conversation that can be objectively observed and measured? Are there predictable causes that will manifest predictable effect? Alternatively, is the conversation socially and interpersonally based? Can the reality of the conversation be understood differently by the attending than by the resident? These questions illustrate how the ontology of a phenomenon can be conceived in different ways—that is, the nature of what the phenomenon *is* can be studied in different ways. *Epistemology* relates to what we consider to be rigorous knowledge about the conversation. We might say that objective, value-free descriptions of the conversation that can be reproduced are rigorous knowledge. Alternatively, we might say that knowledge of the conversation is created by the interactions between the attending and the learner, and so, the knowledge is tied to their subjective experiences of the conversation. As this example illustrates, different ontologies are aligned with different epistemologies; together, they are part of the foundational elements of a *paradigm*—that is, stance the researcher takes when investigating a phenomenon.

### How Does Education Research Differ From Other Types of Research?

Engaging in neurology education research is different from the biomedical models of research. Engaging in neurology education research will likely be more difficult than simply reading up on the existing literature and then building a neurology education study. Why? The reason is that the biomedical models of research that work well in clinical research do not translate well to addressing research questions in medical education research.

To illustrate how medical education research is different, consider a scenario where a neurologist wants to understand whether her approach to teaching junior residents about migraine diagnostic criteria enables these learners to reach more accurate migraine diagnoses in their patients. What are some of the considerations she will attend to if she engages in an experiment-based research design? To know whether it was *her specific educational intervention* that made a difference, the neurologist would need to control (and perhaps even limit) the education the resident cohort received about migraines outside of her educational intervention. Is it possible to exert such control? If it is, is it ethical? How would the neurologist construct a control group? Would the control group receive no migraine education? Alternatively, would we need several participant groups, each receiving different kinds of educational interventions? How many learners does the neurologist need in each group to reach a sufficient sample size? How many patients with migraine would each participant need to see for the neurologist to calculate the effectiveness of her intervention? How many years would it take to collect enough diagnoses to understand the effectiveness of her educational intervention? How will we know that the impact observed in patient diagnoses is related to the educational intervention and not to some other factor arising between the time of her intervention and the date when sufficient patient encounters are recorded? This example highlights how experiment-based research designs from the biomedical models of research (e.g., randomized controlled trials or case-control studies) can lack alignment with a medical education research question and fail to address the multiple contextual factors involved in learning. Instead, we can turn to research approaches from the social sciences that offer rigorous ways of examining the impact of the educational intervention for different individuals.

As this scenario illustrates, the biomedical model of experimentation does not transfer well to medical education questions. Neurology education research needs to be approached from a different orientation. This is not to say that there is no place for clinically and biomedically informed research designs in neurology education; however, the utility of those studies is limited. For example, pretest and post-test studies of educational interventions are informative. Still, the research conducted in the broader field of medical education has illustrated that, regardless of the educational intervention delivered to learners, “if you teach students, they will learn.”^3^ In fact, even when there is no designed intervention, that is, when learners are told to engage in self-directed learning, health professions education learners demonstrate improved KSAs.^4^ While there are aspects of neurology education that can be usefully explored through clinically and biomedically informed study designs, other research designs are needed to answer some of the fundamental research questions that need to be addressed by neurology education research.

Education research questions commonly originate from lived experiences and observations in the process of working and engaging in the education of learners. However, to effectively study and explore education-related observations, we must also contend with the fact that investigations are embedded in environments that are fluctuating, deeply contextual, interpersonal, socially constructed, and full of nuance. Given these considerations, medical education research approaches are often aligned with the social sciences, not the experimental designs of clinical and basic science research. In other words, what works for basic science and clinical research questions does not necessarily work in education research.

## Considering Ontology, Epistemology, and Paradigms as a Starting Point in Developing Neurology Education Research: Case Scenarios

Because medical education research often begins with experiences and observations of teaching trainees, we provide 2 examples of commonplace scenarios in neurology education to illustrate how to recognize the nature of the phenomenon to be studied (ontology) and the kind of knowledge about that phenomenon that can be developed through the research (epistemology).

### Scenario 1: Lumbar Puncture Competence

Performing a lumbar puncture is a foundational skill developed during neurology training. If you are involved in training residents to perform lumbar punctures, you probably have asked yourself, what is involved in getting a trainee to master this technical skill? A neurology education research question about this skill might ask, when is a neurology resident ready to perform a lumbar puncture independently (i.e., unsupervised)?

With this question in mind, reflect on the ontology (nature of the phenomenon) of *lumbar puncture skills*. Lumbar punctures involve engaging with patients' physical bodies to obtain a sample of cerebrospinal fluid. Over the course of years of research, the medical community has studied countless methods and developed best practices for conducting lumbar punctures. In so doing, we have learned the cause-and-effect relationships of certain procedural decisions and techniques that learners need to consider when performing this skill. We can measure a learner's proficiency with this skill by relying on this (ever-evolving) body of evidence. Therefore, for the phenomenon of lumbar puncture skills, there is a physical reality that we can objectively observe to construct descriptions of cause-and-effect relationships. We can identify the variables to observe and measure to predict outcomes of lumbar punctures. While knowledge will continue to evolve, lumbar puncture skills can be labeled, defined, measured, and assessed. We can develop a body of knowledge and a correct (or at least currently favored) technique that learners should master to be considered ready to conduct the procedure independently. If this is the nature of lumbar puncture skills, a neurology education researcher can design a study based on the premise that there is an observable level of proficiency for this procedure that residents need to acquire.

With this reflection on the nature of the phenomenon of study, now reflect on the epistemology (kind of knowledge to develop) in the study of lumbar puncture skills. Because lumbar puncture skills can be objectively observed, measured, and predicted, we want to develop unbiased knowledge about the procedure and the skills residents need to master to be ready to perform the procedure independently. This knowledge should be generalizable so that the skills expected of residents to perform lumbar punctures independently would be the same across many different (if not all) contexts. The researcher might design a study to test the hypothesis that a second-year neurology resident is ready to independently perform lumbar punctures after conducting 5 of these procedures under the supervision of and with guidance from an expert attending physician. The researcher would carefully monitor variables—including, for example, how many procedures need to be conducted, how patient recipients vary, and how much supervision and guidance is needed with each procedure—to determine whether the hypothesis is true or whether it needs to be revised.

This study about lumbar punctures likely feels feasible to many readers because it draws on the same assumptions (i.e., the same paradigm) seen in many clinical and biomedical studies. While the study itself is not a randomized control trial (i.e., a research design we see often in clinical and biomedical studies), it upholds the same expectations about the research—that is, to understand the world, we should engage in objective observation of the cause-and-effect relationships between different variables. While knowledge will continue to evolve, research should generate the most unbiased and generalizable findings possible.

This kind of research adheres to a specific set of assumptions that underpin scientific research (i.e., a paradigm), and it sits within the paradigm of *post-positivist* research (i.e., where scholars study the objective reality to uncover unbiased knowledge about that reality). However, there are other ways of conducting research—ways that are often used in medical education research and social sciences. Let us consider a different scenario that will require us to conduct research from a different set of foundational assumptions (i.e., a different research paradigm).

### Scenario 2: Communicating a New Neurologic Diagnosis

Frequently, a neurologist's work involves communicating neurologic diagnoses to patients. Part of the role of an educator is teaching trainees communication skills, especially challenging communication. A research question that a neurology educator might ask about this situation is what is the best way for residents to communicate a new neurologic diagnosis to patients?

With this question in mind, reflect on the ontology (nature of the phenomenon) of *communicating a new diagnosis to patients*. Effectively communicating a diagnosis to a patient is a deeply subjective experience. While there are some general communication principles that are applicable to all patients when having these difficult conversations, there are individual factors that may affect the conversation. What might be effective communication expectations for some patients (i.e., “give it to me straight, doctor”) will stand in direct contradiction of the expectations held by others (i.e., “break it to me gently, doctor”). The way to give diagnostic news to a geriatric patient and their family might be very different from the approach taken with a pediatric patient and their family. Ethnicity, socioeconomic status, and social expectations of different age groups are just some of the considerations that affect the way *effective* communication can change from one patient to the next. Given the nature of the phenomenon, asking what is *the best way to communicate* is nonsensical. There is no *best* way. Many different ways exist; some will be more effective with some individuals, and others will be more effective with others. Developing a broadly generalizable and objective *truth* about the *best way to communicate a diagnosis* is not possible. Therefore, a different kind of research question is needed, a question that is less absolute in its goal—that is, instead of searching for *the best* approach, we might search for the considerations that patients say meaningfully enable them to comprehend the diagnosis and to feel empowered to make informed decisions about their care plan with their neurology resident. The question needs to be different in its scope—that is, instead of developing a communication approach that is applicable to all patients, we might seek to develop an approach that a specific group of patients favors. Therefore, given the nature of the phenomenon of *communicating a new diagnosis to patients*, a more viable research question the neurology educator might ask is what considerations do young adults with a new diagnosis of Parkinson disease describe as meaningfully enabling them to comprehend their diagnosis and to feel empowered to make informed decisions about their care plan with their neurology resident?

With this reflection on the nature of the phenomenon of study, now reflect on the epistemology (kind of knowledge to develop) and recognize that it differs from the lumbar puncture skills scenario. The researcher could observe residents communicating a new neurologic diagnosis to a patient. Still, those observations will not provide insights into the considerations these patients consider to be meaningfully enabling their understanding and action-taking decisions for their care. Nor can the researcher objectively measure those considerations. Instead, the researcher will need to engage with the patients to solicit their reflections, understandings, and decisions. The researcher will also need to recognize that the data collected will be subjective—that is, insights will be developed when the researcher engages with individual patients, asking open-ended questions that encourage these patients to share their personal experiences. The researcher will, therefore, likely have a semistructured interview script that acts as a set of guiding questions that support the conversation to help the researcher gain insight into the patient's experience. In this way, the researcher and the patient collaborate to co-construct the data. Instead of generating an unbiased data set, the data will be subjectively shaped and the findings will not be broadly generalizable; instead, the research will generate rich, detailed insights about the experiences and perspectives of the specific patients in the study. From these data, the researcher will inductively generate knowledge by recognizing, understanding, and contrasting the data from multiple interviews. The research will not create a defined list of what residents should say to communicate a new neurologic diagnosis; instead, it will generate insights into the foundational considerations that the participants describe as supporting their comprehension and action-taking decisions in important ways.

This kind of research draws on the foundational assumptions (i.e., paradigm) of a different approach to science: *constructivism* (i.e., where reality is constructed and nonobjective and where knowledge is recognized as context-dependent). This is a very different approach to research than the one presented in scenario 1. It is deeply important to remember that *different* should not be equated with *better* or *worse*. Instead, because of the nature of the phenomenon and of the kind of knowledge that can be built about that phenomenon, we need to adopt a different set of assumptions and expectations.

## What Do These Scenarios Show Us About Neurology Education Research?

Understanding and outlining the ontology and epistemology of the phenomenon and determining the paradigmatic orientation of a study are foundational steps to performing high-quality research. Although these formal terms from the philosophy of science may not be explicitly present when thinking of designing a research study, they are the names of the assumptions that uphold the ways of thinking that researchers adopt to conduct research. We realize that this part of the process might seem daunting and inaccessible at first; however, finding clarity about the paradigms, ontology, and epistemology is important because the rigor of a research study is different in different paradigms. We advise seeking help and potential collaboration from colleagues with experience with education research when initially designing education research projects (similar to how you would seek statistical collaboration and guidance for biomedical research projects when necessary).

If we do not take care of these foundational steps at the start of the research, the risk of generating research lacking rigor and meaning is high. For example, if the neurology education researcher tries to bring a post-positivist orientation (as illustrated in the design in scenario 1) to a phenomenon that cannot be objectively observed (like the one in scenario 2), even the best-designed study will fail to generate useful insights. Why? The reason is that the phenomenon cannot be meaningfully understood by working from the assumptions and expectations of that paradigm.

## Practical Considerations for Planning Neurology Education Research

Based on the earlier discussion and scenarios, there are practical steps to consider during the initial stages of development of neurology education research. We suggest attending to these practical considerations early on in the research process (i.e., before engaging in study design) because they influence the subsequent steps and decisions about the research study. This influence can be seen in the methodology of the study, when, for example, choosing a qualitative methodology or a quantitative methodology. Other areas of influence include research designs and the methods for data collection (these subsequent steps in the research process will be covered in future FuNER articles).

1. Identify the phenomenon: What are the phenomena to study?
  - In the first case example, the phenomenon of *lumbar puncture competence* (scenario 1) was studied.
  - In the second case scenario, the phenomenon of *new diagnosis communication* (scenario 2) was studied.
2. Identify the assumptions (paradigmatic orientation): What is the paradigmatic orientation of the study?
  - In the example scenarios, the phenomenon of lumbar puncture competence was studied through a post-positivist paradigm and the phenomenon of new diagnosis communication through a constructivism paradigm.
  - The paradigm from which a phenomenon is studied will shape the kind of knowledge that is developed. For example, in scenario 2, the phenomenon of study is *new diagnosis communication*. As per our discussion earlier, studying communication to create knowledge about the *right way* to communicate with all patients is not feasible. Different populations have different communication needs and expectations. Instead, studying *new diagnosis communication* as a context-specific construction where there is no objective *right answer* will allow us to generate principles about the foundational considerations that need to be attended to when communicating with different populations.
3. Embrace the challenge.
  Answering these questions is not easy at first. There are steps to overcome challenges in defining the phenomena of study, ontological and epistemological considerations, and paradigmatic orientation:
  - Explore the literature around the phenomena of study:
    - Understanding the literature will allow you to know what scholarly conversation you are joining and what a study can potentially add to the conversation through the process of conducting research. This description of how the planned study is situated in the context of other studies in the literature will be a key component to construct the introduction section of an article.
    - High-quality education research studies will include descriptions of the paradigmatic orientations (with their ontological and epistemological considerations) and their alignment with different phenomena of study. Understanding these descriptions will help you make the right choices when planning a research study. A recent article provides an example of a high-quality education study that clearly states their orientation.^5^
  b. Seek help when needed and practice identifying the concepts described in this article when reading the literature:
    - There are many resources available in the medical education literature to learn more about the philosophy of science concepts discussed in this article (e.g., for an in-depth discussion of Philosophy of Science topics, a recent series on the Philosophy of Science published in *Academic Medicine*^6^ can be referred).
    - There are individuals with expertise in health professions education research at most academic institutions that you can reach out for a “consult” or potential collaboration.
    - Next time you are reading the *Neurology: Education* journal (or any education journal), we suggest to practice identifying where in the article the abovementioned questions are addressed (e.g., Is the phenomena of study well-described? Is the paradigmatic orientation clear and aligned with the phenomena of study? Are the ontological and epistemological elements aligned with the paradigmatic orientation?). The more you identify these concepts when reading articles, the more equipped you will be to address them when planning an education research study.

## Conclusions

As this discussion highlights, medical education research challenges the researcher to embrace different ways of thinking about research (i.e., different paradigms in the philosophy of science). Neurology education takes place in the messy reality of clinical contexts and the complex reality of patients' lives; it does not often occur in spaces where confounders can be isolated. Medical education research requires scholars to engage with influential considerations (what might be labeled as variables in some paradigms) in relation to their context, considering the complex relations that exist between the individuals, the place, and the time where the educational experiences occur.

The ontological and epistemological considerations we highlight in the scenarios may be frustrating for researchers trained in, and familiar with, the biomedical research tradition, because, when studying phenomena related to education, there are rarely *right* answers that can be objectively determined and universally generalized. The complexity of medical education research stems from the need to explore phenomena from different paradigmatic orientations—some that dovetail with biomedical research traditions (e.g., post-positivism as illustrated in scenario 1) and some that are incommensurate with that approach (e.g., constructivism as illustrated in scenario 2).

The important thing for neurology education researchers to know—before beginning a research study—is that the nature of the phenomena being explored determines the kinds of answers that research can generate and the kinds of research designs that should be harnessed to develop that knowledge.

## Glossary

KSAs: knowledge, skills, and attitudes

## References

[1] Kuhn TS. The Structure of Scientific Revolutions. University of Chicago Press; 1962:xv, 172 p.

[2] Varpio L, MacLeod A. Philosophy of science series: harnessing the multidisciplinary edge effect by exploring paradigms, ontologies, epistemologies, axiologies, and methodologies. Acad Med. 2020;95(5):686-689. doi:10.1097/ACM.0000000000003142 31876567

[3] Cook DA. If you teach them, they will learn: why medical education needs comparative effectiveness research. Adv Health Sci Educ Theory Pract. 2012;17(3):305-310. doi:10.1007/s10459-012-9381-0 22696095

[4] Murad MH, Coto-Yglesias F, Varkey P, Prokop LJ, Murad AL. The effectiveness of self-directed learning in health professions education: a systematic review. Med Educ. 2010;44(11):1057-1068. doi:10.1111/j.1365-2923.2010.03750.x 20946476

[5] Paton M, Zakeri B, Rowland P, et al. Decision making in continuing professional development organisations during a crisis. Med Educ. 2024;58(6):722-729. doi:10.1111/medu.15265 38105389

[6] Philosophy of Science Series. Accessed July 27, 2023. journals.lww.com/academicmedicine/pages/collectiondetails.aspx?TopicalCollectionId=70.

[7] Rees CE, Crampton PES, Monrouxe LV. Re-visioning academic medicine through a constructionist lens. Acad Med. 2020;95(6):846-850. doi:10.1097/ACM.0000000000003109 31809294

[8] Young ME, Ryan A. Postpositivism in health professions education scholarship. Acad Med. 2020;95(5):695-699. doi:10.1097/ACM.0000000000003089 32345881

